# Psychological health outcomes of traditional Chinese exercises in older adults: a meta-analysis of randomized controlled trials

**DOI:** 10.7717/peerj.20773

**Published:** 2026-02-11

**Authors:** Di Geng, Xiaogang Li, Yan Shi

**Affiliations:** 1 Postdoctoral Research Station in Sports Science, Shanxi University, Taiyuan, China; 2 Department of Physical Education, University of Electronic Science and Technology of China, Chengdu, China; 3 School of Physical Education, Sichuan Normal University, Chengdu, China

**Keywords:** Older adults, Traditional Chinese exercises, Tai Chi, Baduanjin, Psychological health, Depression, Anxiety

## Abstract

**Background:**

In recent years, an increasing number of systematic reviews and meta-analyses have examined the health benefits of traditional Chinese exercises (TCEs). However, most of the reviews and meta-analyses have just focused on their effects on physical function in older adults. This study conducts a meta-analysis of existing randomized controlled trials (RCTs) to investigate the effects of TCEs on psychological health outcomes.

**Methods:**

Five Chinese and English databases were searched from their inception to July 2, 2025. RCTs were included if they used TCE interventions to improve psychological health outcomes in older adults, such as depression, anxiety, subjective well-being, general self-efficacy, and self-esteem. Study screening and data extraction were performed independently by two reviewers at all stages. Meta-analyses were conducted using Rev Man 5.4 and Stata 17.0. Pre-determined subgroups included the type of TCEs, mode of the intervention, duration of the intervention, frequency of exercise, duration of the single exercise session, and type of control condition.

**Result:**

This study included 42 RCTs involving 4,317 participants. The meta-analysis revealed that, compared to control groups, TCEs are more effective in improving depression (SMD = −0.51, 95% CI [−0.72 to −0.29], *p* < 0.00001), anxiety (SMD = −0.39, 95% CI [−0.63 to −0.15], *p* = 0.002), and subjective well-being among older adults (SMD = 1.07, 95% CI [0.18–1.96], *p* = 0.02). TCEs also showed positive effects on general self-efficacy (SMD = 0.63, 95% CI [−0.05 to 1.31], *p* = 0.07) and self-esteem (SMD = 0.54, 95% CI [−0.06 to 1.13], *p* = 0.08), although these effects are not statistically significant. Despite the high heterogeneity in some outcomes, the studies with heterogeneity did not have a significant effect on the results. None of the included studies reported any injuries or adverse events among the participants.

**Conclusion:**

TCEs represent an effective and safe intervention that can significantly reduce depressive and anxiety symptoms among older adults, while also enhancing their subjective well-being. When implementing TCE interventions, it is recommended to adopt a group-based exercise class, with sessions lasting 30 to 50 minutes, conducted 3 to 5 times per week, for a minimum duration of 24 weeks.

**Registration:**

PROSPERO (No. CRD420251067843).

## Introduction

Against the backdrop of an accelerating global population aging, healthy aging is a shared issue faced by every country. However, due to a decline in physical functioning and a shift in social roles, older adults are vulnerable to mental disorders such as anxiety, depression, and stress-related disorders. The prevalence of anxiety symptoms among older adults ranges from 15% to 52.3%, while that of depression reaches as high as 35.1% (Bryant, Jackson & Ames, 2008; Kilpatrick et al., 2022). Compared with younger individuals, older adults with psychological disorders tend to show poorer emotion regulation and cognitive functioning. They also frequently present with comorbid physical conditions such as hypertension, diabetes, and coronary artery disease (Kandasamy et al., 2025; Lin & Von Korff, 2008; Marin et al., 2011). Psychological disorders not only significantly impair quality of life but also impose a heavy burden on families and society. According to the Global Burden of Disease (GBD), the age-standardized incidence rate (ASIR) and age-standardized disability-adjusted life years (ASDR) for mental disorders increased by 15.23% and 17.28% between 1990 and 2021. These trends indicate that psychological disorders continue to rank among the top ten global health burdens (Fan et al., 2025). Psychological health is not simply the absence of mental illness; rather, it refers to a positive and adaptive psychological state in which individuals can function effectively and realize their physical and cognitive potential (World Health Organization, 2005). Studies have shown that such positive states are associated with a lower prevalence of age-related diseases (Grossman et al., 2020). Therefore, it is essential for governments to increase investment in psychological health services and to provide older adults with broader support and treatment options. 

Exercise, as a non-pharmacological intervention, can alleviate psychological problems to some extent and positively influence health beliefs. A cross-sectional study of adults showed a significant association between physical activity and self-reported psychological health burden. The psychological health burden was assessed based on participants’ reports over the past month (Chekroud et al., 2018). Both organized and individual forms of exercise were shown to provide psychological benefits compared to no exercise (Seino et al., 2019). Despite the proven benefits of physical activity, most older adults do not engage in regular exercise. A survey on exercise adherence in fall-prevention programs among older adults found low participation rates. Only 12% engaged in strength training, and just 6% participated in balance training (Merom et al., 2012). The effectiveness of exercise-based rehabilitation depends on several structural factors, including frequency, intensity, duration, and mode of exercise. Inappropriate exercise prescriptions can reduce adherence to interventions (Rivera-Torres, Fahey & Rivera, 2019). With aging, the musculoskeletal system undergoes structural and functional decline due to physiological degeneration and age-related diseases. Therefore, it is essential to provide exercise options that are tailored to the physical capabilities of older adults to improve adherence to exercise interventions.

Traditional Chinese exercises (TCEs) are fitness practices guided by the principles of traditional Chinese medicine. Common types of TCEs include Tai Chi, Baduanjin, Liuzijue, Wuqinxi, and Yijinjing. Compared to other forms of physical activity, TCEs are characterized by low intensity, prolonged duration, and slow rhythm, making them particularly suitable for older adults and individuals with chronic conditions (Guo et al., 2016). In recent years, an increasing number of systematic reviews and meta-analyses have examined the health benefits of TCEs. However, most of them have just focused on their effects on physical function in older adults (Xie, Guo & Wang, 2024; Wang et al., 2022). TCEs contain prominent mindfulness components, as they require practitioners to focus their attention on the present movements and breathing, without being distracted by external stimuli. In addition, TCEs are often performed in group settings, providing participants with opportunities for social interaction. According to social cognitive theory, environmental factors can stimulate and shape cognition, and there is a close relationship between cognitive function and mental health. From this theoretical perspective, participation in TCEs may alter environmental factors for older adults, thereby promoting positive cognition and mental well-being. To assess the psychological benefits of TCEs in older populations, Dong et al. (2025) conducted a meta-analysis in 2025 that investigated the clinical effects of TCEs on alleviating psychological disorders in older adults. Although their findings demonstrated that TCEs significantly reduced symptoms of anxiety and depression, several limitations remain in their study. First, Dong et al. (2025) did not explore the impact of TCEs on positive psychological outcomes, such as subjective well-being, self-efficacy, and self-esteem, which limits the understanding of TCEs’ overall benefits for psychological health in older adults. Second, they did not analyze different types or modes of TCE interventions as potential sources of heterogeneity in the results. Third, while their review assessed the effectiveness of TCEs in reducing depression and anxiety, they did not report whether any adverse events occurred. Therefore, the safety of TCEs as a psychological health intervention for older adults remains to be further evaluated.

To address the limitations of previous systematic reviews and meta-analyses, this study conducted a meta-analysis based on data from randomized controlled trials (RCTs) to evaluate the effectiveness of TCEs compared with control conditions in improving psychological health outcomes among older adults. We hypothesized that TCEs would improve psychological outcomes influenced by social participation and support. In addition, we hypothesize that intervention type, mode, duration, frequency, and period may serve as moderating variables in the relationship between traditional Chinese exercises (TCEs) and mental health in older adults. Therefore, the second aim of this study is to explore the optimal intervention strategy of TCEs for improving mental health among the elderly.

## Method

### Protocol and registration

In accordance with the PRISMA guidelines, the protocol was prospectively registered on the PROSPERO platform (registration number: CRD420251067843).

### Information sources and search

We systematically searched five electronic databases, including PubMed, Web of Science, Embase, EBSCOhost, and China National Knowledge Infrastructure (CNKI), from their inception to July 2, 2025. The search strategy included a combination of relevant terms and their synonyms: (i) traditional Chinese exercise, Qigong, Tai Ji, Baduanjin, Wuqinxi, Yijinjing, Liuzijue; (ii) aged; (iii) mental health, affect, emotion, depression, anxiety, psychological well-being, self-efficacy, self concept; and (iv) trial, randomized, and experimental. In addition, the following search filters were applied: “clinical trial”. A detailed example of the search strategy used for EBSCOhost is presented in Table 1.

### Inclusion and exclusion criteria

Two reviewers independently screened the literature. Any disagreements were resolved through discussion with a third author. The inclusion criteria were as follows: (i) Participants: older adults (mean age ≥ 60 years), regardless of whether they had a diagnosed mental disorder; (ii) Intervention: TCEs (Tai Chi, Baduanjin, Liuzijue, Wuqinxi, and Yijinjing); (iii) Comparison: active or non-active control conditions, with active interventions excluding any components of TCEs; (iv) Outcomes: at least one of the following psychological outcomes: depression, anxiety, subjective well-being, general self-efficacy, or self-esteem; (v) Study design: RCTs.

The exclusion criteria were as follows: (i) full text not available; (ii) insufficient statistical data for extraction (*e.g.*, sample size, means, standard deviations); (iii) combination of TCE interventions with non-TCE modes of physical activity; (iv) significant baseline differences between the intervention and control groups; (v) gray literature, such as technical reports, dissertations, and conference abstracts; (vi) studies not published in English or Chinese.

### Data extraction

To minimize potential bias, two experienced researchers (DG and XL) performed data collection in July 2025. The extracted data included the following: (i) bibliographic information (first author’s surname and year of publication); (ii) participant demographics (sample source, sample size, and age); (iii) characteristics of the TCE intervention. If a study did not provide detailed descriptions of the intervention protocol, we traced the references cited in the article to identify the intervention type; (iv) control conditions; (v) outcome measures. For studies with multiple control groups, results for each group were extracted separately. Any discrepancies between the two reviewers were resolved by consultation with the corresponding author (YS), who verified the data and made the final decision.

**Table 1:** Search strategy in EBSCOhost.

**Search number**	**Search term**
**#1**	SU (tai chi OR taiji OR qigong OR liuzijue OR wuqinxi OR yijinjing OR baduanjin OR traditional exercise OR chinese traditional exercise OR traditional chinese exercise OR chinese exercise)
**#2**	SU (anxi* OR angst OR nervousness OR hypervigilance OR depress* OR affect* OR mood* OR mental health OR mental hygiene OR emotion* OR feeling* OR regret* OR stress OR well-being OR wellness OR self-efficacy OR self-esteem OR self concept)
**#3**	SU (Aged or old* or elder* or senior)
**#4**	TX (Randomized controlled trial or trial or randomized or randomised or placebo)
**#5**	#1 and #2 and #3 and #4

### Quality appraisal

Two reviewers independently assessed the methodological quality of the included studies using the Physiotherapy Evidence Database (PEDro) scale. The PEDro scale consists of 11 items; except for the first item (eligibility criteria), the remaining 10 items are used to calculate the total score (Maher et al., 2003). Each study received a total score ranging from 0 to 10 based on its methodological quality. Following previous studies (Geng, Li & Sun, 2025; Morales et al., 2024), we categorized study quality into three levels: high (score ≥6), moderate (score 4–5), and low (score ≤3). Any disagreements regarding quality assessment were reviewed and resolved by the corresponding author (YS).

### Statistical analysis

Data were analyzed using Review Manager software (Version 5.4) and Stata software (Version 17.0). The effect size was calculated using the mean and standard deviation of post-intervention outcomes. Because all outcome variables in the included studies were continuous and the measurement tools varied, standardized mean differences with 95% confidence intervals (CIs) were used to estimate effect sizes. A result was considered statistically significant when *p* < 0.05, indicating a significant difference between the TCE and control groups. Heterogeneity across studies was assessed using the *Q* statistic and the *I^2^* statistic, with *I^2^* values of 25%, 50%, and 75% indicating low, moderate, and high heterogeneity, respectively. When heterogeneity was not substantial (*p* ≥ 0.10, I^2^ < 50%), a fixed-effects model (inverse variance method) was applied; otherwise, a random-effects model was used. Subgroup analyses were conducted to examine the influence of potential moderators. Predefined subgroups included TCE type, intervention mode, intervention duration, frequency, session length, and type of control condition. For outcomes with high heterogeneity (*I^2^* ≥ 75%), sensitivity analyses were performed using the one-by-one removal method to explore the sources of heterogeneity and assess the robustness of the meta-analysis results. When ten or more studies were included in a given analysis, publication bias was evaluated using a combination of funnel plot inspection (qualitative) and Egger’s regression test (quantitative).

## Results

### Search and selection

**Figure 1:**
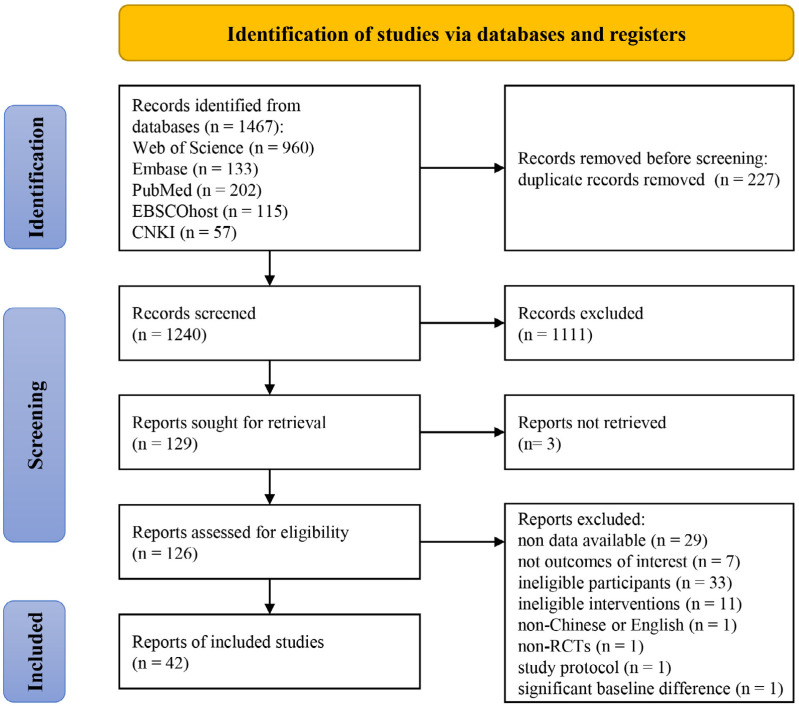
Flow diagram of the search results and study selection.

A total of 1,467 records were initially identified through database searches. Duplicate records (*n* = 227) were removed using EndNote X9 reference management software. Titles and abstracts of the remaining records were screened according to predefined inclusion criteria, resulting in the exclusion of 1,111 irrelevant studies. Subsequently, full-text screening of the remaining 126 records resulted in the exclusion of 84 articles for various reasons: unavailable data (*n* = 29), outcome indicators mismatch (*n* = 7), ineligible participants (*n* = 33), intervention content mismatch (*n* = 11), non-English or Chinese articles (*n* = 1), non-RCTs (*n* = 1), study protocol (*n* = 1), significant baseline difference (*n* = 1). Final, 42 RCTs were included in the meta-analysis (Cai et al., 2023; Carcelén-Fraile et al., 2022; Chan, Yu & Choi, 2017; Chen et al., 2024; Cheng et al., 2012; Chou et al., 2004; Ge et al., 2021; He et al., 2024a; He et al., 2024b; Hsu et al., 2016; Huang et al., 2019; Huang et al., 2025; Irwin et al., 2014; Jing et al., 2018; Kerkez & Erci, 2024; Kilpatrick et al., 2022; Krause-Sorio et al., 2024; Kutner et al., 1997; Lam et al., 2012; Lee et al., 2019; Liang et al., 2020; Liu et al., 2018; Luo et al., 2023; Ma et al., 2018; Martínez et al., 2014; Moon et al., 2020; Noradechanunt, Worsley & Groeller, 2017; Qiu et al., 2024; Redwine et al., 2020; Roswiyani et al., 2019; Solianik et al., 2021; Su, Wang & Meng, 2021; Taylor-Piliae et al., 2014; Tousignant et al., 2012; Tsang et al., 2003; Tsang et al., 2006; Tsang et al., 2013; Wang et al., 2010; Wang et al., 2023; Wu et al., 2017; Xiao et al., 2023; Ying, Shen & Wang, 2019). The study selection process is illustrated in Fig. 1. There was strong agreement between the reviewers for the screening records and full texts (Kappa: 0.82).

### Characteristics of the included studies

The included studies involved between 14 and 472 participants, with a total sample size of 4,317. Participants represented a variety of populations, including individuals with chronic physical conditions, sedentary older adults, patients with mild dementia, depression, Parkinson’s disease, and stroke survivors. Four types of TCE interventions were identified: Tai Chi (*n* = 27), Baduanjin (*n* = 12), Liuzijue (*n* = 2), and Yijinjing (*n* = 1). The interventions were delivered in three modes: group-based exercise classes (*n* = 31), self-practice (*n* = 3), and a combination of both (*n* = 8). The duration of the interventions ranged from 4 to 52 weeks, with 12 weeks being the most common. For group-based classes, the duration per session ranged from 20 to 120 min, while for self-practice, session length ranged from 5 to 60 min. The frequency of group classes ranged from once every two weeks to five times per week, most commonly three times per week. Self-practice was performed at frequencies ranging from once every other day to twice daily. The included studies employed different types of control conditions. In 14 studies, active controls were used, such as recreational activities (*e.g.*, playing mahjong, arts), cognitive training, health education, or other forms of physical exercise. In contrast, 23 studies used non-active controls, such as routine care, usual rehabilitation, placebo, waitlist, newspaper reading, or maintenance of normal lifestyle. Additionally, five studies included both active and non-active control groups. The basic characteristics of the included studies are summarized in Table 2.

**Table 2:** Characteristics of eligible studies.

**Study**	**Source of sample**	**Sample size (** ***n*** **)**	**Age (years)**	**Intervention (type)**		**Mode, frequency and duration**	**Outcomes**
				T	C		
Cai et al. (2023)	Chronic physical illness	47	67.79 ± 5.59	Baduanjin	Cognitive training	Exercise class: 60 min × 2 times per week × 12 weeks	Subjective well-being
Carcelén-Fraile et al. (2022)	Postmenopausal women	117	69.73 ± 6.44	Baduanjin	Daily activities	Exercise class: 60 min × 2 times per week, 12 weeks	Anxiety, depression
Chan, Yu & Choi (2017)	Hidden elderly	46	77.31 ± 7.46	Tai chi	Usual care	Exercise class: 60 min × 2 times per week × 12 weeks self-practice: 30 min × 1 time per day × 12 weeks	Anxiety, depression, self-esteem
Chen et al. (2024)	Chronic obstructive pulmonary disease	472	61.9 ± 10.66	Baduanjin	1. usual care 2. conventional rehabilitation	Self-practice: 30 min × 10 times per week × 24 weeks	Anxiety, depression
Cheng et al. (2012)	Mild dementia	36	81.8 ± 6.36	Tai chi	1. placebo 2. mahjong	Exercise class: 60 min × 3 times per week × 12 weeks	Depression
Chou et al. (2004)	Depressive disorders	14	72.6 ± 4.2	Tai chi	Wait-list	Exercise class: 45 min × 3 times per week × 12 weeks	Depression
Ge et al. (2021)	Pre-frail elderly people	65	71.56 ± 6.16	Tai chi	Usual care	Exercise class: 60 min × 3 times per week × 8 weeks	Depression
He et al. (2024a)	Sleep disturbances	38	69.24 ± 4.72	Tai chi	Usual treatments	Exercise class: 60 min × 3 times per week × 4 weeks	Anxiety, depression
He et al. (2024b)	Sleep disturbances	152	67.68 ± 4.98	Tai chi	Low-intensity physical exercise	Exercise class: 60 min × 3 times per week × 4 weeks	Anxiety, depression
Hsu et al. (2016)	Long-term care residents	60	81.25 ± 8.12	Tai chi	Usual care	Exercise class: 40 min × 3 times per week × 26 weeks	Depression
Huang et al. (2019)	Mild dementia	80	81.9 ± 6.01	Tai chi	Usual treatments	Exercise class: 20 min × 3 times per week × 40 weeks	Depression
Huang et al. (2025)	Depressive symptoms	240	69.58 ± 6.95	Tai chi	Aerobic exercise	Exercise class: 60 min × 3 times per week × 12 weeks	Depression
Irwin et al. (2014)	Insomnia	123	65.55 ± 6.97	Tai chi	1. sleep seminar 2. cognitive training	Exercise class: 120 min × 3 times per week × 16 weeks	Depression
Jing et al. (2018)	Elderly housebound	79	75.16 ± 6.04	Baduanjin	Cognitive training	Exercise class: 60–90 min × 1 time every two weeks × 12 weeks	Depression
Kerkez & Erci (2024)	General elderly	114	71.34 ± 4.14	Tai chi	Non-intervention	Exercise class: 35–40 min × 3 times per week × 6 weeks	Depression
Kilpatrick et al. (2022)	Depressive disorders	40	67.62 ± 6.47	Tai chi	Health education	Exercise class: 60 min × 1 time per week × 12 weeks self-practice: 20 min × 1 time per day × 12 weeks	Depression
Krause-Sorio et al. (2024)	Depressive disorders	49	67.98 ± 6.12	Tai chi	Health education	Exercise class: 60 min × 1 time per week × 12 weeks self-practice: 20 min × 1 time per day × 12 weeks	Anxiety, depression
Kutner et al. (1997)	Healthy older adults	130	76.2	Tai chi	1. health education 2. balance training	Exercise class: 45 min × 2 times per week × 15 weeks	Self-esteem
Lam et al. (2012)	Cognitive decline	389	77.82 ± 6.48	Tai chi	Stretching and toning exercise	Exercise class: 30 min × 3 times per week × 48 weeks	Depression
Lee et al. (2019)	Chronic physical illness	30	>60	Baduanjin	Cognitive training	Exercise class: 2 times per week × 12 weeks	Anxiety, depression, well-being
Liang et al. (2020)	Elderly housebound	48	71.88 ± 4.44	Tai chi	1. exercise advice 2. exercise snacking	Self-practice: 5 min × 2 times per day × 4weeks	Anxiety, depression
Liu et al. (2018)	Depressive disorders	60	61.31 ± 3.92	Tai chi	Normal lifestyle	Exercise class: 60 min × 3 times per week × 24 weeks	Depression
Luo et al. (2023)	moderate to severe COPD	226	67.74 ± 6.95	Tai chi	conventional therapy, exercise advice, and health education	Self-practice: 30 min × 5 times per week × 52 weeks	Anxiety, depression
Ma et al. (2018)	hypertension	158	69.98 ± 10.52	Tai chi	Usual care	Exercise class: 90 min × 2 times per week × 5 weeks self-practice: 60 min/ session, 3–5 session/week, 24 weeks	Depression
Martínez et al. (2014)	care rehabilitation facility	58	74.3 ± 8.2	Liuzijue	Usual care and rehabilitation	Exercise class: 90 min × 2 times per week × 4 weeks	Depression
Qiu et al. (2024)	private medical centre	200	60-66	Tai chi	Non-intervention	Exercise class: 1 time per week × 24 weeks	Depression
Moon et al. (2020)	Parkinson’s disease	17	66.14 ± 6.58	Liuzijue	Sham qigong	Exercise class: 45–60 min × 1 time per week × 12 weeks, self-practice: 15–20 min × 2 times per day × 12 weeks	Anxiety, depression
Noradechanunt, Worsley & Groeller (2017)	Sedentary elderly	39	66.6 ± 6.7	Tai chi	1. exercise advice 2. Thai Yoga	Exercise class: 90 min × 2 times per week × 12 weeks self-practice: 20 min × 1 time every other day × 12 weeks	Depression
Redwine et al. (2020)	Heart failure	69	65 ± 10	Tai chi	1. usual treatments 2.resistance band exercise	Exercise class: 45–60 min × 3 times per week × 16 weeks, self-practice: 10–20 min × 1 time per non-class day × 12 weeks	Depression
Roswiyani et al. (2019)	Nursing homes	195	73.44 ± 9.13	Baduanjin	1. non-intervention 2. art activities	Exercise class: 90 min × 2 times per week × 8 weeks	Depression
Solianik et al. (2021)	General elderly	30	60–78	Tai chi	Non-intervention	Exercise class: 60 min × 2 times per week × 10 weeks	Anxiety, depression
Su, Wang & Meng (2021)	Memory complaints	65	64.89 ± 6.41	Baduanjin	Gymnastics practice	Exercise class: 60 min × 5 times per week × 12 weeks	Self-efficacy
Taylor-Piliae et al. (2014)	Stroke survivors	145	69.9 ± 10	Tai chi	1. usual care 2. strength and movement exercises	Exercise class: 60 min × 3 times per week × 12 weeks	Depression
Tousignant et al. (2012)	High risk for a fall	152	79.9 ± 6.2	Tai chi	Usual rehabilitation	Exercise class: 60 min × 2 times per week × 15 weeks	Self-efficacy
Tsang et al. (2003)	Chronic physical illnesses	50	74.67 ± 9.03	Baduanjin	Usual rehabilitation	Exercise class: 60 min × 2 times per week × 12 weeks	Depression
Tsang et al. (2006)	Depressive disorders and chronic medical illnes	82	82.37 ± 7.01	Baduanjin	Newspaper reading	Exercise class: 45–60 min × 3 times per week × 16 weeks self-practice: 15 min × 1 time per day × 16 weeks	Depression, self-efficacy, subjective well-being
Tsang et al. (2013)	Depressive disorders and chronic medical illnes	38	80.11 ± 5.63	Baduanjin	Newspaper reading	Exercise class: 45 min × 3 times per week × 12 weeks	Depression, self-efficacy,
Wang et al. (2010)	Cerebral vascular disorder	34	77.06 ± 10.95	Tai chi	Usual rehabilitation	Exercise class: 50 min × 1 time per week × 12 weeks	Anxiety, depression
Wang et al. (2023)	General elderly	80	60–69	Tai chi	Normal lifestyle	Exercise class: 60 min × 4 times per week × 12 weeks	Subjective well-being
Wu et al. (2017)	High risk for a fall	120	65–80	Baduanjin	Normal lifestyle	Exercise class: 2 times per day × 4weeks	Anxiety
Xiao et al. (2023)	General elderly	56	68.85 ± 5.02	Yijinjing	Normal lifestyle	Exercise class: 40 min × 3 times per week × 8 weeks	Self-esteem
Ying, Shen & Wang (2019)	Insomnia	74	71.93 ± 3.67	Baduanjin	Walking exercise	Exercise class: 60 min × 5 times per week × 12 weeks	Anxiety

**Notes.**

T TCEs group C Control group COPD Chronic obstructive pulmonary disease

### Quality appraisal

The PEDro scores of the 42 included studies ranged from 3 to 8, with an average score of 5.98, indicating an overall high level of methodological quality (Table 3). Among them, 28 studies were rated as high quality (scores of 6–8), while the remaining 13 were considered to be of moderate quality (scores of 4–5). It should be noted that, due to the nature of physiotherapy interventions, blinding was not implemented in most of the included studies.

**Table 3:** Methodological quality of the included studies.

**Study**	**EC**	**RA**	**CA**	**SAB**	**SB**	**TB**	**AB**	**DR**	**ITA**	**BC**	**PM**	**TS** ^a^	**OSQ**
Cai et al. (2023)	1	1	0	1	0	0	1	0	1	1	1	6	High
Carcelén-Fraile et al. (2022)	1	1	1	1	0	0	0	1	0	1	1	6	High
Chan, Yu & Choi (2017)	1	1	1	1	0	0	1	1	0	0	1	6	High
Chen et al. (2024)	1	1	0	1	0	1	1	1	1	1	1	8	High
Cheng et al. (2012)	1	1	0	1	0	0	0	0	1	0	1	4	Moderate
Chou et al. (2004)	1	1	0	1	0	0	1	0	1	1	1	6	High
Ge et al. (2021)	1	1	0	1	0	0	1	1	0	1	1	6	High
He et al. (2024a)	1	1	0	1	0	0	1	1	1	1	1	7	High
He et al. (2024b)	1	1	1	1	0	0	1	1	1	1	1	8	High
Hsu et al. (2016)	1	1	1	1	0	0	0	1	1	1	1	7	High
Huang et al. (2019)	1	1	1	1	0	0	0	1	0	1	1	6	High
Huang et al. (2025)	1	1	1	1	0	0	1	1	1	1	1	8	High
Irwin et al. (2014)	1	1	1	1	0	0	1	1	1	1	1	8	High
Jing et al. (2018)	1	1	0	1	0	0	0	1	0	1	1	5	Moderate
Kerkez & Erci (2024)	1	1	0	1	0	0	0	1	0	1	1	5	Moderate
Kilpatrick et al. (2022)	1	1	0	1	0	0	0	0	0	1	1	4	Moderate
Krause-Sorio et al. (2024)	1	1	0	1	1	0	1	0	1	1	1	7	High
Kutner et al. (1997)	1	1	0	1	0	0	0	0	0	0	1	3	Low
Lam et al. (2012)	1	1	0	1	0	0	1	0	0	1	1	5	Moderate
Lee et al. (2019)	0	1	1	1	0	0	1	0	1	1	1	7	High
Liang et al. (2020)	1	1	1	1	0	0	1	0	0	0	1	5	Moderate
Liu et al. (2018)	1	1	1	1	0	0	1	1	1	0	1	7	High
Luo et al. (2023)	1	1	1	1	0	0	1	1	0	0	1	6	High
Ma et al. (2018)	1	1	1	1	0	0	0	0	0	1	1	5	Moderate
Martínez et al. (2014)	1	1	1	1	0	0	1	1	0	1	1	7	High
Qiu et al. (2024)	1	1	0	1	0	0	0	1	1	1	1	6	High
Moon et al. (2020)	1	1	1	1	1	0	1	0	0	1	1	7	High
Noradechanunt, Worsley & Groeller (2017)	1	1	0	1	0	0	1	0	0	1	1	5	Moderate
Redwine et al. (2020)	1	1	1	1	0	0	1	0	0	1	1	6	High
Roswiyani et al. (2019)	1	1	1	1	0	0	0	1	1	1	1	7	High
Solianik et al. (2021)	1	1	0	1	0	0	1	1	1	1	1	7	High
Su, Wang & Meng (2021)	1	1	0	1	0	0	0	0	0	1	1	4	Moderate
Taylor-Piliae et al. (2014)	1	1	0	1	0	0	1	1	1	1	1	7	High
Tousignant et al. (2012)	1	1	1	1	0	0	1	0	0	1	1	6	High
Tsang et al. (2003)	0	1	0	1	0	0	0	0	0	1	1	4	Moderate
Tsang et al. (2006)	1	1	0	1	1	0	1	0	0	1	1	6	High
Tsang et al. (2013)	1	1	1	1	0	0	1	0	1	1	1	7	High
Wang et al. (2010)	1	1	0	1	0	0	1	1	0	1	1	6	High
Wang et al. (2023)	1	1	0	1	0	0	0	0	1	1	1	5	Moderate
Wu et al. (2017)	1	1	0	1	0	0	0	0	1	1	1	5	Moderate
Xiao et al. (2023)	0	1	1	1	0	0	0	1	1	1	1	7	High
Ying, Shen & Wang (2019)	0	1	0	1	0	0	0	0	0	1	1	4	Moderate
Mean score												5.98	

**Notes.**

Yes = 1; No = 0.

EC eligibility criteria RA random allocation CA concealed allocation SAB similar at baseline SB subject blinded TB therapist blinded AB assessor blinded DR dropout rate (<15%) ITA intention-to-treat analysis BC between-group comparison PM points measures TS total score OSQ overall study quality

a A score out of 10 is determined from the number of criteria that are satisfied, except that scale item 1 is not used to generate the total score.

### Primary outcomes

#### Depression

**Figure 2:**
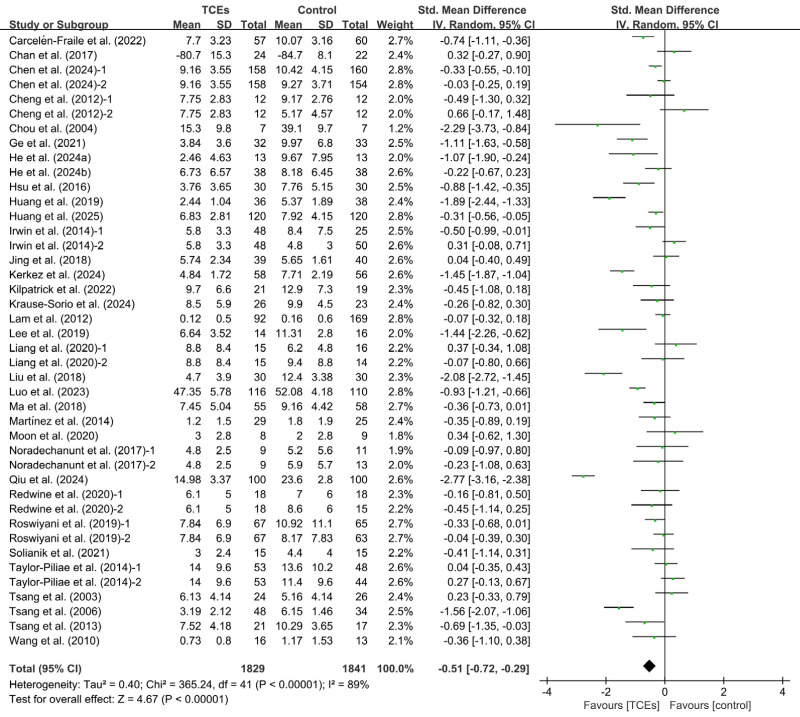
Forest plot of the effects of TCEs on depression.

A total of 34 studies reported the effects of TCEs on depression. Among them, eight studies included two control groups, resulting in 42 comparisons. Due to substantial heterogeneity among the included studies (*I^2^* = 89%, *p* < 0.00001), a random-effects model was applied. The meta-analysis showed that TCEs significantly reduced depressive symptoms in older adults compared with control conditions, and the difference was statistically significant (SMD = −0.51, 95% CI [−0.72 to −0.29], *p* < 0.00001) (Fig. 2). Sensitivity analysis indicated that the exclusion of any single study did not lead to substantial changes in the overall effect size or heterogeneity.

#### Anxiety

**Figure 3:**
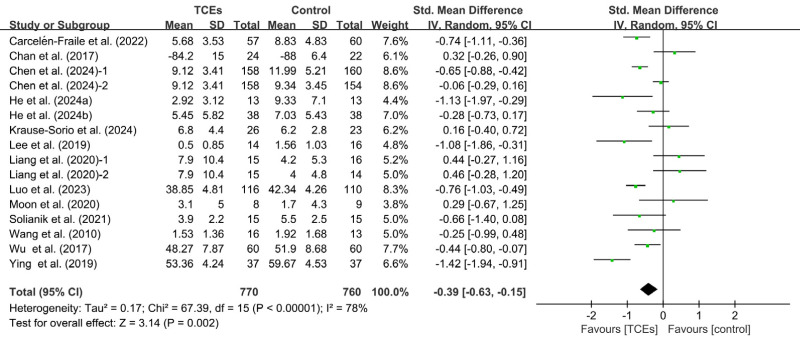
Forest plot of the effects of TCEs on anxiety.

Fourteen studies reported the effects of TCEs on anxiety. Among them, two studies included two control groups, resulting in 16 comparisons. Heterogeneity among the studies was high (*I^2^* = 78%, *p* < 0.00001), a random-effects model was applied. The meta-analysis showed that TCEs significantly reduced anxiety symptoms in older adults compared with control conditions, and the difference was statistically significant (SMD = −0.39, 95% CI [−0.63 to −0.15], *p* = 0.002) (Fig. 3). Sensitivity analysis indicated that excluding any single study did not result in notable changes in the overall effect size or heterogeneity.

### Secondary outcome

#### Subjective well-being

**Figure 4:**
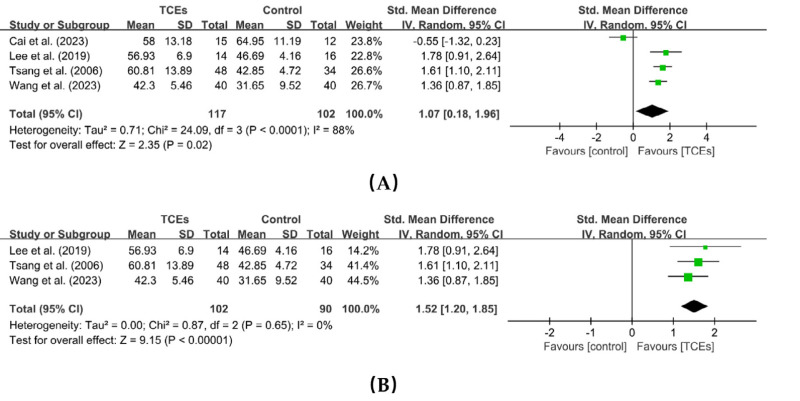
Forest plot of the effects of TCEs on subjective well-being. (A) Before a sensitivity analysis. (B) After a sensitivity analysis.

Four studies reported the effects of TCEs on subjective well-being, involving a total of 219 participants. The pooled results showed a large heterogeneity among the studies (*I^2^* = 88%, *p* < 0.0001), a random-effects model was applied. The meta-analysis showed that TCEs significantly improved subjective well-being in older adults (SMD = 1.07, 95% CI [0.18–1.96], *p* = 0.02). Sensitivity analysis identified one study as the primary source of heterogeneity. After removing this study (Cai et al., 2023), heterogeneity was reduced to 0%, and the effect size remained largely unchanged (SMD = 1.52, 95% CI [1.20–1.85], *p* < 0.00001) (Fig. 4). Compared with the other three studies, the number of participants who provided measurable outcomes in the study by Cai et al. (2023) was very small (total <30), which may have increased the risk of sampling error and resulted in findings that may not adequately reflect the true population effects. Therefore, we speculate that differences in the number of participants with available outcome data may be the source of the high heterogeneity.

#### General self-efficacy

**Figure 5:**
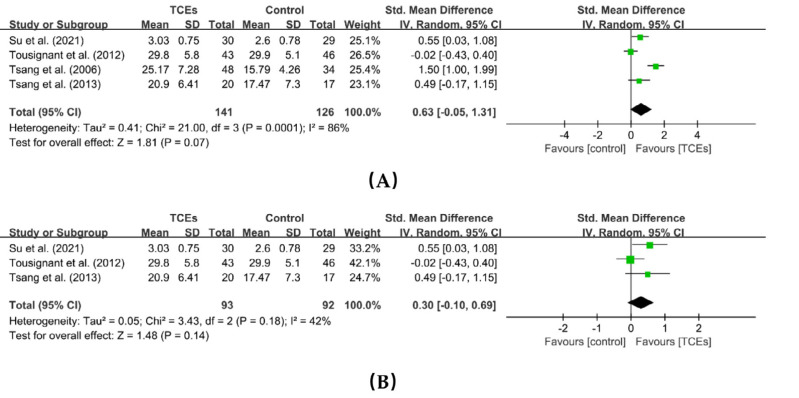
Forest plot of the effects of TCEs on general self-efficacy. (A) Before a sensitivity analysis. (B) After a sensitivity analysis.

Four studies reported the effects of TCEs on general self-efficacy, involving a total of 267 participants. The meta-analysis indicated that TCEs improved general self-efficacy in older adults; however, the difference was not statistically significant (SMD= 0.63, 95% CI [−0.05 to 1.31], *p* = 0.07). Considerable heterogeneity was observed among the included studies (*I^2^* = 86%, *p* = 0.0001). One study was heterogeneous following a sensitivity analysis. After excluding this study (Tsang et al., 2006), heterogeneity decreased to 42%, while the effect size remained largely unchanged (SMD = 0.30, 95% CI [−0.10 to 0.69], *p* = 0.14) (Fig. 5). Unlike the single-intervention approach used in the other three studies, Tsang et al. (2006) employed a mixed mode combining supervised exercise sessions with self-directed practice. We suggest that this difference may have contributed to the observed high heterogeneity.

#### Self-esteem

**Figure 6:**
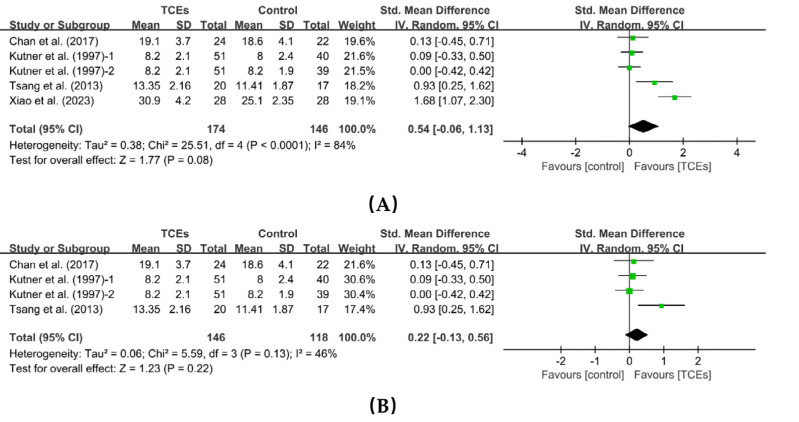
Forest plot of the effects of TCEs on self-esteem. (A) Before a sensitivity analysis. (B) After a sensitivity analysis.

Four studies reported the effects of TCEs on self-esteem. One study included two control groups, resulting in five comparisons. The meta-analysis showed that TCEs improved self-esteem in older adults; however, the difference was not statistically significant (SMD = 0.54, 95% CI [−0.06 to 1.13], *p* = 0.08). There was a large heterogeneity of results (*I^2^* = 84%, *p* < 0.0001). One study was heterogeneous following a sensitivity analysis. After eliminating this study (Xiao et al., 2023), heterogeneity decreased from 84% to 46%, and the effect size remained largely unchanged (SMD = 0.22, 95% CI [−0.13 to 0.56], *p* = 0.22) (Fig. 6). After excluding the study by Xiao et al. (2023) the heterogeneity decreased. We suggest that this may be due to differences in intervention duration across studies. Xiao et al. (2023) implemented a short-term intervention (<12 weeks), whereas the remaining studies employed medium-term interventions (≥12 weeks to <24 weeks).

### Subgroup analysis

The meta-analyses for subjective well-being, general self-efficacy, and self-esteem included only a few studies. Because the sources of high heterogeneity were identified through sensitivity analysis, subgroup analyses were conducted only for depression and anxiety. Subgroup analyses were performed based on the following variables: type of TCEs (Baduanjin, Tai Chi, Liuzijue); intervention mode (group-based classes, self-practice, or a combination of both); session duration (5–20 min/session, 30–50 min/session, ≥60 min/session); intervention frequency (<3 times/week, 3–5 times/week, >7 times/week); intervention duration (short term: <12 weeks; medium term: ≥12 to <24 weeks; long term: ≥24 weeks); and type of control condition (active, non-active). It should be noted that for studies combining group-based classes and self-practice, if the session duration or frequency differed between the two modes, the study was excluded from subgroup analysis for that specific variable. However, if the two modes differed in intervention duration, the longer duration was used for subgroup classification. Additionally, for studies that included multiple control groups, data from each control group were extracted separately for subgroup analysis and were counted toward the total number of studies and participants.

Subgroup analysis based on the type of TCEs revealed no statistically significant differences between groups for either depression or anxiety. However, the magnitude of effects varied across TCE types: Tai Chi was associated with greater improvement in depression (SMD = −0.55, 95% CI [−0.84 to −0.26], *p* = 0.0002), while Baduanjin showed a more pronounced effect in reducing anxiety (SMD = −0.67, 95% CI [−1.04 to −0.31], *p* = 0.0003). Subgroup analysis based on intervention mode showed no significant group differences for depression, but a significant difference was observed for anxiety (subgroup difference *p* = 0.0003). Compared with other intervention modes, group-based exercise classes had a more substantial positive impact on both depression (SMD = −0.63, 95% CI [−0.94 to −0.33], *p* < 0.0001) and anxiety (SMD = −0.72, 95% CI [−1.02 to −0.42], *p* < 0.00001) (Table 4).

**Table 4:** Summary of subgroup analysis results.

**Outcome**	**Category of variables**	**Studies (n)**	**Participants (n)**	***SMD*** ** (95%** ***CIs*** **)**	***p*** **-value (overall effect)**	***I*** ^*2*^ ** value (%)**	***p*** **-value (heterogeneity)**	***p*** **-value** **(group differences)**
Depression	Exercise type							
	Baduanjin	10	1,288	−0.43 (−0.72 to −0.14)	0.003^*^	83	<0.00001^*^	0.47
	Tai chi	30	2,311	−0.55 (−0.84 to −0.26)	0.0002^*^	90	<0.00001^*^	
	Liuzijue	2	71	−0.12 (−0.76 to 0.51)	0.70	33	0.22	
	Exercise mode							
	Exercise class	27	2,296	−0.63 (−0.94 to −0.33)	<0.0001^*^	91	<0.00001^*^	0.26
	Self-practice	5	916	−0.26 (−0.66 to 0.15)	0.21	86	<0.00001^*^	
	Combined	10	458	−0.33 (−0.69 to 0.03)	0.08	70	0.0004^*^	
	Single time duration							
	5–20 min	3	134	−0.54 (−2.00 to 0.92)	0.47	93	<0.00001^*^	0.31
	30–50 min	9	1,372	−0.65 (−1.00 to −0.30)	0.0003^*^	88	<0.00001^*^	
	≥60 min	18	1,476	−0.32 (−0.56 to −0.08)	0.009^*^	79	<0.00001^*^	
	Exercise frequency							
	<3 times/week	10	780	−0.51 (−1.17 to 0.15)	0.13	94	<0.00001^*^	0.03^*^
	3–5 times/week	18	1,671	−0.65 (−0.97 to −0.33)	<0.0001^*^	89	<0.00001^*^	
	>7 times/week	4	690	−0.11 (−0.36 to 0.14)	0.37	47	0.13	
	Durations of exercise							
	Short term	10	687	−0.47 (−0.83 to −0.12)	0.009^*^	79	<0.00001^*^	0.06
	Medium term	23	1,359	−0.29 (−0.51 to −0.06)	0.01^*^	74	<0.00001^*^	
	Long term	9	1,624	−1.01 (−1.58 to −0.45)	0.0004^*^	96	<0.00001^*^	
	Type of control							
	Active	17	1,485	−0.17 (−0.39 to 0.05)	0.13	73	<0.00001^*^	0.004^*^
	Non-active	25	2,185	−0.74 (−1.06 to −0.42)	<0.00001^*^	91	<0.00001^*^	
Anxiety	Exercise type							
	Baduanjin	6	971	−0.67 (−1.04 to −0.31)	0.0003^*^	85	<0.00001^*^	0.07
	Tai chi	9	542	−0.19 (−0.57 to 0.18)	0.31	74	0.0002^*^	
	Liuzijue	1	17	0.29 (−0.67 to 1.25)	0.56	NA	NA	
	Exercise mode							
	Exercise class	8	502	−0.72 (−1.02 to −0.42)	<0.00001^*^	57	0.02^*^	0.0003^*^
	Self-practice	5	916	−0.22 (−0.63 to 0.20)	0.30	87	<0.00001^*^	
	Combined	3	112	0.24 (−0.13 to 0.62)	0.20	0	0.92	
	Single time duration							
	5–20 min	2	60	0.45 (−0.06 to 0.97)	0.09	0	0.97	0.0007^*^
	30–50 min	4	885	−0.45 (−0.84 to −0.07)	0.02^*^	85	0.0002^*^	
	≥60 min	5	323	−0.82 (−1.24 to −0.41)	0.0001^*^	66	0.02^*^	
	Exercise frequency							
	<3 times/week	4	206	−0.70 (−0.98 to −0.42)	<0.00001^*^	0	0.49	0.03^*^
	3–5 times/week	4	402	−0.86 (−1.32 to −0.40)	0.0003^*^	74	0.009^*^	
	>7 times/week	5	810	−0.15 (−0.53 to 0.23)	0.44	82	0.0002^*^	
	Durations of exercise							
	Short term	6	312	−0.26 (−0.67 to 0.14)	0.20	63	0.02^*^	0.75
	Medium term	7	362	−0.42 (−0.94 to 0.11)	0.12	81	<0.0001^*^	
	Long term	3	856	−0.49 (−0.92 to −0.06)	0.03^*^	90	<0.0001^*^	
	Type of control							
	Active	7	515	−0.38 (−0.87 to 0.11)	0.13	84	<0.00001^*^	0.99
	Non-active	9	1,015	−0.38 (−0.66 to −0.11)	0.006^*^	71	0.0005^*^	

**Notes.**

NA not applicable

* *p* < 0.05.

Subgroup analysis based on session duration found significant positive effects of interventions lasting 30-50 min or more than 60 min per session on both depression and anxiety. In contrast, sessions lasting 5–20 min had no significant effect on either outcome. Subgroup analysis based on exercise frequency revealed significant group differences for both depression (subgroup difference *p* = 0.03) and anxiety (subgroup difference *p* = 0.03). The impact of exercise frequency varied across outcomes: a frequency of 3–5 times per week significantly improved depression, whereas both <3 times/week and 3–5 times/week were associated with significant improvements in anxiety, with comparable effect sizes (Table 4).

Subgroup analysis based on intervention duration revealed no significant group differences for either depression or anxiety. All three durations of TCE interventions significantly improved depression, with long-term interventions demonstrating the most pronounced effects. However, for anxiety, only long-term interventions produced statistically significant improvements. Subgroup analysis based on control conditions showed significant group differences for depression (subgroup difference *p* = 0.004), but not for anxiety. TCE interventions produced significant improvements in both depression and anxiety under non-active control conditions, whereas no significant effects were observed under active control conditions (Table 4).

### Publication bias

Among the five outcomes analyzed, only depression and anxiety were examined in ten or more studies. Therefore, publication bias was assessed for these two outcomes using funnel plot inspection and Egger’s regression test. The Egger’s test yielded *p*-values of 0.37 for depression and 0.64 for anxiety, indicating no significant evidence of publication bias. However, because fewer than ten studies were available for subjective well-being, general self-efficacy, and self-esteem, we were unable to reliably evaluate publication bias for these three outcomes. Consequently, the possibility of underlying publication bias cannot be ruled out.

### Adverse events

Among the 42 included RCTs, 13 reported on adverse events. Of these, 12 studies stated that no injuries or adverse reactions occurred in the TCE group. One study reported that a small number of participants experienced transient palpitations during TCEs practice, which resolved after rest.

## Discussion

As far as we know, no prior meta-analysis has comprehensively evaluated the effects of TCE interventions on a broad range of psychological health outcomes in older adults. This study addressed this gap by including multiple psychological outcomes, such as depression, anxiety, subjective well-being, general self-efficacy, and self-esteem. It also synthesized evidence exclusively from randomized controlled trials, which constitute the highest level of evidence. Therefore, our findings provide more comprehensive and robust evidence supporting the effectiveness of TCEs in improving psychological health in older adults. In this meta-analysis of 42 RCTs involving 4,317 older participants, we found that TCE interventions had a beneficial effect on psychological health. This positive effect was reflected in two main aspects. First, TCEs significantly alleviated depression and anxiety, which are the most prevalent psychological health problems in older adults. Second, TCEs were also associated with improvements in positive psychological outcomes, including subjective well-being, general self-efficacy, and self-esteem. Based on the available evidence, TCE interventions have been most commonly applied in the treatment of depression and anxiety. Therefore, these two outcomes were the focus of in-depth analyses in the present study.

The meta-analysis results for depression indicated that TCEs significantly alleviated depressive symptoms in older adults. According to Cohen’s classification of effect sizes (Cohen, 1988), our findings showed a moderate effect size (*d* =  − 0.51) for TCE interventions on depression. This result is largely consistent with the findings of the previous meta-analysis by Dong et al. (2025). Several clinical practice guidelines have acknowledged the psychological health benefits of aerobic exercise and recommend it as a treatment option for alleviating depressive symptoms (Carneiro et al., 2018). Compared with general aerobic exercises (*e.g.*, brisk walking), TCEs have been shown to exert stronger effects on increasing gray matter volume in brain regions such as the left medial frontal gyrus, left superior temporal gyrus, and right middle temporal gyrus (Cui et al., 2019). Gray matter is closely associated with many health conditions, including depression, and its increased volume may have positive implications for cognitive functioning and psychological health (Ancelin et al., 2019). In our study, Tai Chi and Baduanjin demonstrated stronger antidepressant effects than Liuzijue, with effect sizes of *d* = −0.55 and *d* = −0.43, respectively. Furthermore, interventions delivered through group-based exercise classes produced more pronounced effects on depression compared to the other two modes. Although the combined intervention mode also included group-based classes, these accounted for a smaller proportion of the intervention, with self-practice being the dominant mode. Previous studies have shown that social engagement and participation in group activities are closely related to better psychological health outcomes in older adults (Giummarra et al., 2007). Group-based interventions may enhance social connectedness and facilitate interpersonal interactions within the exercise classes, thereby amplifying the antidepressant effects of TCEs.

In this study, we found that TCEs significantly reduced anxiety levels in older adults, with a moderate effect size (*d* = −0.39). A previous meta-analysis reported that aerobic exercise was not effective in treating anxiety disorders when compared with control conditions such as waitlist or placebo, cognitive behavioral therapy, or psychoeducation (Bartley, Hay & Bloch, 2013). Unlike general aerobic exercises, TCEs incorporate a non-judgmental mindfulness component, requiring practitioners to maintain inner calm and focused attention, while eliminating distractions in pursuit of mind–body integration (Dong et al., 2024). Existing literature suggests that the combined effects of low-to-moderate intensity physical activity and mindfulness meditation may contribute to reduced levels of cortisol and adrenaline, and enhanced secretion of endorphins, all of which are hormones closely linked to anxiety and stress regulation (Zheng et al., 2018). Our subgroup analysis further showed that session durations of 30–50 min or more than 60 min were more effective in reducing anxiety than shorter sessions lasting 5–20 min. The effect sizes of TCE interventions performed 3–5 times per week and fewer than 3 times per week were comparable, and both were more significant than those of interventions performed seven or more times per week. In most exercise prescriptions, higher frequency is often associated with shorter session durations. According to the *WHO Guidelines on Physical Activity and Sedentary Behaviour*, older adults are recommended to engage in at least 150–300 min of moderate-intensity aerobic activity per week, along with at least three sessions of multicomponent physical activity to achieve substantial health benefits (World Health Organization, 2020). Based on these findings, we recommend engaging in TCEs 3 to 5 times per week, with each session lasting 30 to 50 min, as an optimal strategy for reducing anxiety in older adults.

In addition to alleviating common mental disorders, we found that TCEs also improved positive psychological outcomes in older adults, including subjective well-being (*d* = 1.07), general self-efficacy (*d* = 0.63), and self-esteem (*d* = 0.54). Lower levels of psychological well-being have been associated with the presence of chronic physical conditions, which are highly prevalent among older adults (Mehnert et al., 1990). Unfortunately, high-quality clinical trials investigating the impact of TCEs on well-being in this population remain limited, with only four studies meeting our predefined inclusion criteria. Notably, a study by Cai et al. (2023) found that TCEs did not significantly enhance subjective well-being compared with cognitive training. This null finding may be due to the active control condition (cognitive training) being more beneficial than the control conditions used in the other three studies. Besides, although our findings showed improvements in general self-efficacy following TCE interventions, and the effect sizes reached moderate levels, these effects were not statistically significant. Previous studies have examined the effects of psychosocial interventions on general self-efficacy among older adults, and their findings are consistent with those of the present stud, namely that general self-efficacy does not show significant improvement (Barnes & Markham, 2018; Connor et al., 2019). It is generally believed that general self-efficacy is shaped through long-term effort and experience, and is influenced by the interaction between accumulated experience and psychological regulation (FitzGerald, Wells & Ellis, 2022). Such traits may be difficult to change through interventions, especially among older adults. Therefore, it is not yet possible to make any predictions regarding the effectiveness of TCEs in supporting or enhancing general self-efficacy. Similarly, our findings indicate that the improvement in self-esteem associated with TCEs is not statistically significant. Previous research has reported that physical exercise yields greater improvements in self-esteem among younger individuals compared with older adults, possibly because younger adults tend to have better physical fitness (Amesberger et al., 2019; Awick et al., 2017). Besides, self-esteem reflects an individual’s self-evaluation of their social roles. It is considered a psychological component of personality-related self-regulation, and personality is a relatively stable psychological trait (Erdle et al., 2010). This may explain why, despite moderate effect sizes, improvements in self-esteem were not statistically significant.

Several outcomes in this study exhibited substantial heterogeneity. We employed sensitivity analyses and subgroup analyses to explore the potential sources of this heterogeneity. For studies that were identified as contributing to heterogeneity, we compared the standardized mean differences (SMDs) before and after exclusion. The sensitivity analyses indicated that the direction of the SMDs remained consistent across all outcomes, suggesting that the results are robust. However, for depression and anxiety, the sensitivity analyses did not identify any specific studies responsible for the observed heterogeneity. Therefore, we conducted subgroup analyses to explore potential moderators and assess the influence of different types, modes, and dosages of TCE interventions on these outcomes in older adults. The subgroup analyses revealed that, in the case of depression, variability in intervention frequency and control conditions contributed to the observed heterogeneity. For anxiety, the heterogeneity appeared to stem from differences in intervention mode, duration of each session, and exercise frequency. In recent years, numerous studies have explored exercise prescriptions aimed at improving mental health (Escobar-Roldan, Babyak & Blumenthal, 2021; Kleemann et al., 2020). Researchers generally agree that the effects of physical activity on mental disorders vary substantially depending on the exercise “dose”. In other words, beneficial psychological effects can only be achieved when appropriate frequency, intensity, and duration of exercise are maintained. For example, one study found that patients with major depressive disorder who engaged in supervised exercise three times per week experienced improvements comparable to those receiving pharmacotherapy with sertraline after four months, whereas exercising fewer than three times per week did not yield similar benefits (Blumenthal et al., 2007). This helps explain why our subgroup analyses revealed clinical heterogeneity arising from differences in intervention protocols across the included studies.

### Strengths and limitations

One strength of this study is that it included only randomized controlled trials. Given the overall high methodological quality of the included studies, the findings can be interpreted from a causal perspective. Furthermore, this analysis synthesized a variety of positive psychological outcomes, including subjective well-being, general self-efficacy, and self-esteem. This offers a more comprehensive evaluation and confirmation of the psychological health benefits of TCEs in older adults compared to previous meta-analyses. Another strength lies in the consideration of several key moderating factors, such as the type of TCEs, intervention mode, and type of control condition. These factors provide clearer insights for future study design and the development of more targeted interventions.

This meta-analysis has several limitations that should be considered in future research. First, the present analysis included only studies published in English and Chinese, which may limit the generalizability of our findings. Broader literature searches that include studies published in other languages are warranted to ensure more comprehensive coverage of available evidence. Second, most of the studies included in the analysis were conducted in China, which may further limit the generalizability of our findings. Therefore, we recommend that future research be conducted across more diverse populations from different countries or ethnic groups to determine whether these effects vary across demographic contexts. Third, although we examined the effects of different types of TCEs on depression and anxiety, the vast majority of the included studies focused on Tai Chi and Baduanjin. As a result, the effects of other traditional exercises such as Yijinjing and Wuqinxi on common mental disorders remain unclear. Finally, due to the nature of physical activity interventions, most studies did not implement blinding for participants or instructors, which may have introduced performance or detection bias and potentially exaggerated the observed effects.

### Practical implications

This study highlights the positive effects of TCEs on promoting psychological health among older adults, particularly in reducing depression and anxiety and enhancing subjective well-being. In light of these findings, it is recommended that public health authorities actively promote the beneficial role of TCEs in improving mental health and enhancing well-being, thereby increasing social acceptance and recognition of TCE-based interventions. Policymakers should also incorporate TCEs into mental health prevention and rehabilitation programs for older adults, making them an important strategy for supporting healthy aging. In addition, community centers and long-term care institutions should strengthen collaboration with sports associations to organize regular group-based exercise sessions for older adults, focusing on practices such as Tai Chi, Baduanjin, or Liu Zi Jue. Meanwhile, instructors in the field of TCEs should consider how to improve older adults’ exercise experience. For example, by integrating intelligent exercise devices or virtual reality-based platforms (*e.g.*, exergames) to increase enjoyment and thereby enhance adherence. Findings related to potential moderators may also provide valuable insights to guide the design and implementation of future TCE interventions. Accordingly, when prescribing TCEs for older adults, healthcare professionals should tailor the duration, frequency, and intervention cycle according to each individual’s physical and psychological condition.

## Conclusion

Despite several limitations, our findings are largely consistent with the initial hypotheses. The results indicate that TCE-based interventions, such as Tai Chi and Baduanjin, can effectively reduce depressive and anxiety symptoms among older adults and generate significant improvements in subjective well-being. In addition, we identified several moderating factors, including intervention format, duration, frequency, and the length of each exercise session, that influence the relationship between TCEs and these psychological outcomes. Based on the current evidence, when using TCEs to improve depression, anxiety, and subjective well-being in older adults, group-based exercise formats are recommended. Each session should last 30 to 50 min, be conducted three to five times per week, and continue for at least 24 weeks. However, due to the limited availability of long-term follow-up data, the long-term effectiveness of TCE interventions remains uncertain. Therefore, future studies should incorporate extended follow-up periods to assess the long-term efficacy, safety, and sustained psychological benefits of TCE-based interventions.

##  Supplemental Information

10.7717/peerj.20773/supp-1 Supplemental Information 1 PRISMA checklist

10.7717/peerj.20773/supp-2 Supplemental Information 2 Systematic Review and/or Meta-Analysis Rationale

10.7717/peerj.20773/supp-3 Supplemental Information 3 The complete dataset used for the meta-analysis in Microsoft Excel format

## Additional Information and Declarations

### Data Availability

The following information was supplied regarding data availability: The raw data are available in the Supplementary File.

## References

[ref-1] Amesberger G, Finkenzeller T, Müller E, Würth S (2019). Aging-related changes in the relationship between the physical self-concept and the physical fitness in elderly individuals. Scandinavian Journal of Medicine & Science in Sports.

[ref-2] Ancelin ML, Carrière I, Artero S, Maller J, Meslin C, Ritchie K, Ryan J, Chaudieu I (2019). Lifetime major depression and grey-matter volume. Journal of Psychiatry & Neuroscience.

[ref-3] Awick EA, Ehlers D, Fanning J, Phillips SM, Wójcicki T, Mackenzie MJ, Motl R, McAuley E (2017). Effects of a home-based DVD-delivered physical activity program on self-esteem in older adults: results from a randomized controlled trial. Psychosomatic Medicine.

[ref-4] Barnes CJ, Markham C (2018). A pilot study to evaluate the effectiveness of an individualized and cognitive behavioural communication intervention for informal carers of people with dementia: the talking sense programme: individualized communication cbt for dementia carers. International Journal of Language & Communication Disorders.

[ref-5] Bartley CA, Hay M, Bloch MH (2013). Meta-analysis: aerobic exercise for the treatment of anxiety disorders. Progress in Neuro-Psychopharmacology & Biological Psychiatry.

[ref-6] Blumenthal JA, Babyak MA, Doraiswamy PM, Watkins L, Hoffman BM, Barbour KA, Herman S, Craighead WE, Brosse AL, Waugh R, Hinderliter A, Sherwood A (2007). Exercise and pharmacotherapy in the treatment of major depressive disorder. Psychosomatic Medicine.

[ref-7] Bryant C, Jackson H, Ames D (2008). The prevalence of anxiety in older adults: methodological issues and a review of the literature. Journal of Affective Disorders.

[ref-8] Cai SH, Tsang HWH, Lu EY, Leung MKW, Siu DCH, Leung SY, Au FLY, Cheung WM, Jensen MP (2023). Enhancing subjective well-being through qigong: a randomized controlled trial in older adults in Hong Kong with chronic physical illness. Alternative Therapies in Health and Medicine.

[ref-9] Carcelén-Fraile MD, Aibar-Almazán A, Martínez-Amat A, Jiménez-García JD, Brandao-Loureiro V, García-Garro PA, Fábrega-Cuadros R, Rivas-Campo Y, Hita-Contreras F (2022). Qigong for mental health and sleep quality in postmenopausal women: a randomized controlled trial. Medicine.

[ref-10] Carneiro LF, Mota MP, Schuch F, Deslandes A, Vasconcelos-Raposo J (2018). Portuguese and Brazilian guidelines for the treatment of depression: exercise as medicine. Brazilian Journal of Psychiatry.

[ref-11] Chan AWK, Yu DSF, Choi KC (2017). Effects of tai chi qigong on psychosocial well-being among hidden elderly, using elderly neighborhood volunteer approach: a pilot randomized controlled trial. Clinical Interventions in Aging.

[ref-12] Chekroud SR, Gueorguieva R, Zheutlin AB, Paulus M, Krumholz HM, Krystal JH, Chekroud AM (2018). Association between physical exercise and mental health in 1.2 million individuals in the USA between 2011 and 2015: a cross-sectional study. Lancet Psychiatry.

[ref-13] Chen Y, Zhang P, Dong ZM, Zhu YA, Liu YA, Qiao C, Zhang N, Jiang YX, Chen B (2024). Effect of Baduanjin exercise on health and functional status in patients with chronic obstructive pulmonary disease: a community-based, cluster-randomized controlled trial. NPJ Primary Care Respiratory Medicine.

[ref-14] Cheng ST, Chow PK, Yu ECS, Chan ACM (2012). Leisure activities alleviate depressive symptoms in nursing home residents with very mild or mild dementia. American Journal of Geriatric Psychiatry.

[ref-15] Chou KL, Lee PWH, Yu ECS, Macfarlane D, Cheng YH, Chan SSC (2004). Effect of Tai Chi on depressive symptoms amongst Chinese older patients with depressive disorders: a randomized clinical trial. International Journal of Geriatric Psychiatry.

[ref-16] Cohen J (1988). Statistical power analysis for the behavioral-sciences perceptual and motor skills.

[ref-17] Connor KI, Cheng EM, Barry F, Siebens HC, Lee ML, Ganz DA, Mittman BS, Connor MK, Edwards LK, McGowan MG, Vickrey BG (2019). Randomized trial of care management to improve parkinson disease care quality. Neurology.

[ref-18] Cui L, Yin HC, Lyu SJ, Shen QQ, Wang Y, Li XJ, Li J, Li YF, Zhu LN (2019). Tai Chi Chuan *vs* general aerobic exercise in brain plasticity: a multimodal MRI study. Scientific Reports.

[ref-19] Dong CY, Liu RY, Li R, Huang ZY, Sun SY (2024). Effects of traditional chinese exercises on glycemic control in patients with type 2 diabetes mellitus: a systematic review and meta analysis of randomized controlled trials. Sports Medicine.

[ref-20] Dong YJ, Pang D, Xiang J, Chao GD, Kuang XQ (2025). Exploring the benefits of traditional Chinese exercises (Tai Chi and Qigong) on the anxiety and depression of older adults: a systematic review and meta-analysis. Medicine.

[ref-21] Erdle S, Irwing P, Rushton JP, Park J (2010). The general factor of personality and its relation to self-esteem in 628 640 internet respondents. Personality and Individual Differences.

[ref-22] Escobar-Roldan ID, Babyak MA, Blumenthal JA (2021). Exercise prescription practices to improve mental health. Journal of Psychiatric Practice.

[ref-23] Fan Y, Fan A, Yang Z, Fan DM (2025). Global burden of mental disorders in 204 countries and territories, 1990–2021: results from the global burden of disease study 2021. BMC Psychiatry.

[ref-24] FitzGerald J, Wells YD, Ellis JM (2022). Psychosocial modification of general self-efficacy in older adults: a restricted review. Australasian Journal on Ageing.

[ref-25] Ge YJ, Liu H, Wu QW, Chen AJ, Gao ZP, Xing FM, Liu GT (2021). Effects of a short eight Tai Chi-forms for the pre-frail elderly people in senior living communities. Physiotherapy Theory and Practice.

[ref-26] Geng D, Li XG, Sun GT (2025). The effectiveness of exercise interventions in the improvement of sleep in older adult people: a meta-analysis. Frontiers in Public Health.

[ref-27] Giummarra MJ, Haralambous B, Moore K, Nankervis J (2007). The concept of health in older age: views of older people and health professionals. Australian Health Review.

[ref-28] Grossman JT, Frumkin MR, Rodebaugh TL, Lenze EJ (2020). mHealth assessment and intervention of depression and anxiety in older adults. Harvard Review of Psychiatry.

[ref-29] Guo YC, Shi HY, Yu DH, Qiu PX (2016). Health benefits of traditional Chinese sports and physical activity for older adults: a systematic review of evidence. Journal of Sport and Health Science.

[ref-30] He JL, Chan SHW, Chung RCK, Tsang HWH (2024b). Effect of combined Tai Chi and repetitive transcranial magnetic stimulation for sleep disturbance in older adults: a randomized controlled trial. Journal of Psychiatric Research.

[ref-31] He JL, Chan SHW, Lin JX, Tsang HWH (2024a). Integration of tai chi and repetitive transcranial magnetic stimulation for sleep disturbances in older adults: a pilot randomized controlled trial. Sleep Medicine.

[ref-32] Hsu CY, Moyle W, Cooke M, Jones C (2016). Seated Tai Chi *versus* usual activities in older people using wheelchairs: a randomized controlled trial. Complementary Therapies in Medicine.

[ref-33] Huang CJ, Li ZL, Chan EHW, Li YF, Hao P, Wang XD, Yao XS (2025). Effects of Tai Chi versus general aerobic exercise on depressive symptoms and serum lipid levels among older persons with depressive symptoms: a randomized controlled study. Journal of Lightwave Technology.

[ref-34] Huang NY, Li WJ, Rong XJ, Champ M, Wei L, Li M, Mu HY, Hu YQ, Ma ZJ, Lyu JH (2019). Effects of a modified Tai Chi program on older people with mild Dementia: a randomized controlled trial. Journal of Alzheimer’s Disease.

[ref-35] Irwin MR, Olmstead R, Carrillo C, Sadeghi N, Breen EC, Witarama T, Yokomizo M, Lavretsky H, Carroll JE, Motivala SJ, Bootzin R, Nicassio P (2014). Cognitive behavioral therapy *vs.* Tai Chi for late life insomnia and inflammatory risk: a randomized controlled comparative efficacy trial. Sleep.

[ref-36] Jing LW, Jin YP, Zhang XL, Wang FL, Song YY, Xing FM (2018). The effect of Baduanjin qigong combined with CBT on physical fitness and psychological health of elderly housebound. Medicine.

[ref-37] Kandasamy G, Subramani T, Almanasef M, Orayj K, Shorog E, Alshahrani AM, Alsaab A, Alshahrani ZM, Palayakkodan S (2025). Mental health and hypertension: assessing the prevalence of anxiety and depression and their associated factors in a tertiary care population. Frontiers in Public Health.

[ref-38] Kerkez M, Erci B (2024). The effect of moving meditation exercise on depression and sleep quality of the elderly a randomized controlled study. Holistic Nursing Practice.

[ref-39] Kilpatrick LA, Siddarth P, Milillo MM, Krause-Sorio B, Ercoli L, Narr KL, Lavretsky H (2022). Impact of Tai Chi as an adjunct treatment on brain connectivity in geriatric depression. Journal of Affective Disorders.

[ref-40] Kleemann E, Bracht CG, Stanton R, Schuch FB (2020). Exercise prescription for people with mental illness: an evaluation of mental health professionals’ knowledge, beliefs, barriers, and behaviors. Brazilian Journal of Psychiatry.

[ref-41] Krause-Sorio B, Siddarth P, Milillo MM, Kilpatrick L, Ercoli L, Narr KL, Lavretsky H (2024). Grey matter volume predicts improvement in geriatric depression in response to Tai Chi compared to health education. International Psychogeriatrics.

[ref-42] Kutner NG, Barnhart H, Wolf SL, McNeely E, Xu TS (1997). Self-report benefits of Tai Chi practice by older adults. Journals of Gerontology Series B-Psychological Sciences and Social Sciences.

[ref-43] Lam LCW, Chau RCM, Wong BML, Fung AWT, Tam CWC, Leung GTY, Kwok TCY, Leung TYS, Ng SP, Chan WM (2012). A 1-year randomized controlled trial comparing mind body exercise (Tai Chi) with stretching and toning exercise on cognitive function in older Chinese adults at risk of cognitive decline. Journal of the American Medical Directors Association.

[ref-44] Lee P, Cai SH, Lu EY, Ng BFL, Jensen MP, Tsang HWH (2019). Qigong reduces depressive symptoms of taiwanese elderly with chronic physical illness: a randomized controlled trial. Journal of Alternative and Complementary Medicine.

[ref-45] Liang IJ, Perkin OJ, McGuigan PM, Thompson D, Western MJ (2020). Feasibility and acceptability of home-based exercise snacking and Tai Chi snacking delivered remotely to self-isolating older adults during COVID-19. Journal of Aging and Physical Activity.

[ref-46] Lin EHB, Von Korff M (2008). Mental disorders among persons with diabetes-results from the world mental health surveys. Journal of Psychosomatic Research.

[ref-47] Liu J, Xie HH, Liu M, Wang ZB, Zou LY, Yeung AS, Hui SSC, Yang Q (2018). The effects of Tai Chi on heart rate variability in older chinese individuals with depression. International Journal Environment Research and Public Health.

[ref-48] Luo CF, Jiang HJ, Li HW, Chi XY (2023). Effects of Tai Chi on patients with moderate to severe COPD in stable phase. Medicine.

[ref-49] Ma CH, Zhou W, Tang QB, Huang SL (2018). The impact of group-based Tai chi on health-status outcomes among community-dwelling older adults with hypertension. Heart & Lung.

[ref-50] Maher CG, Sherrington C, Herbert RD, Moseley AM, Elkins M (2003). Reliability of the PEDro scale for rating quality of randomized controlled trials. Physical Therapy.

[ref-51] Marin MF, Lord C, Andrews J, Juster RP, Sindi S, Arsenault-Lapierre G, Fiocco AJ, Lupien SJ (2011). Chronic stress, cognitive functioning and mental health. Neurobiology of Learning and Memory.

[ref-52] Martínez N, Martorell C, Espinosa L, Marasigan V, Domènech S, Inzitari M (2014). Impact of Qigong on quality of life, pain and depressive symptoms in older adults admitted to an intermediate care rehabilitation unit: a randomized controlled trial. Aging Clinical and Experimental Research.

[ref-53] Mehnert T, Krauss HH, Nadler R, Boyd M (1990). Correlates of life satisfaction in those with disabling conditions. Rehabilitation Psychology.

[ref-54] Merom D, Pye V, Van der Ploeg H, Sherrington C, Lord S, Bauman A (2012). Prevalence and correlates of participation in fall prevention exercise/physical activity by older adults. Preventive Medicine.

[ref-55] Moon S, Sarmento CVM, Steinbacher M, Smirnova IV, Colgrove Y, Lai SM, Lyons KE, Liu W (2020). Can Qigong improve non-motor symptoms in people with Parkinson’s disease—a pilot randomized controlled trial?. Complementary Therapies in Clinical Practice.

[ref-56] Morales JS, Del Río EA, Valenzuela PL, Martinez-de Quel O (2024). Physical activity and cognitive performance in early childhood: a systematic review and meta-analysis of randomized controlled trials. Sports Medicine.

[ref-57] Noradechanunt C, Worsley A, Groeller H (2017). Thai Yoga improves physical function and well-being in older adults: a randomised controlled trial. Journal of Science and Medicine in Sport.

[ref-58] Qiu T, Zhang GH, Zhou FL, Jiang H (2024). Application of virtual reality to enhance therapeutic Tai Chi for depression in elderly people. Acta Psychologica.

[ref-59] Redwine LS, Pung MA, Wilson K, Bangen KJ, Delano-Wood L, Hurwitz B (2020). An exploratory randomized sub-study of light-to-moderate intensity exercise on cognitive function, depression symptoms and inflammation in older adults with heart failure. Journal of Psychosomatic Research.

[ref-60] Rivera-Torres S, Fahey TD, Rivera MA (2019). Adherence to exercise programs in older adults: informative report. Gerontology and Geriatric Medicine.

[ref-61] Roswiyani R, Hiew CH, Witteman CLM, Satiadarma MP, Spijker J (2019). Art activities and qigong exercise for the well-being of older adults in nursing homes in Indonesia: a randomized controlled trial. Aging & Mental Health.

[ref-62] Seino S, Kitamura A, Tomine Y, Tanaka I, Nishi M, Taniguchi Y, Yokoyama Y, Amano H, Fujiwara Y, Shinkai S (2019). Exercise arrangement is associated with physical and mental health in older adults. Medicine & Sscience in Sports & Exercise.

[ref-63] Solianik R, Mickeviciene D, Zlibinaite L, Cekanauskaite A (2021). Tai Chi improves psychoemotional state, cognition, and motor learning in older adults during the COVID-19 pandemic. Experimental Gerontology.

[ref-64] Su H, Wang HN, Meng LN (2021). The effects of Baduanjin exercise on the subjective memory complaint of older adults A randomized controlled trial. Medicine.

[ref-65] Taylor-Piliae RE, Hoke TM, Hepworth JT, Latt LD, Najafi B, Coull BM (2014). Effect of Tai Chi on physical function, fall rates and quality of life among older stroke survivors. Archives of Physical Medicine and Rehabilitation.

[ref-66] Tousignant M, Corriveau H, Roy PM, Desrosiers J, Dubuc N, Hébert R, Tremblay-Boudreault V, Beaudoin AJ (2012). The effect of supervised Tai Chi intervention compared to a physiotherapy program on fall-related clinical outcomes: a randomized clinical trial. Disability and Rehabilitation.

[ref-67] Tsang HWH, Fung KMT, Chan ASM, Lee G, Chan F (2006). Effect of a qigong exercise programme on elderly with depression. International Journal of Geriatric Psychiatry.

[ref-68] Tsang HWH, Mok CK, Yeung YTA, Chan SYC (2003). The effect of Qigong on general and psychosocial health of elderly with chronic physical illnesses: a randomized clinical trial. International Journal of Geriatric Psychiatry.

[ref-69] Tsang HWH, Tsang WWN, Jones AYM, Fung KMT, Chan AHL, Chan EP, Au DWH (2013). Psycho-physical and neurophysiological effects of qigong on depressed elders with chronic illness. Aging & Mental Health.

[ref-70] Wang CY, Liang JL, Si YH, Li ZY, Lu AM (2022). The effectiveness of traditional Chinese medicine-based exercise on physical performance, balance and muscle strength among older adults: a systematic review with meta-analysis. Aging Clinical and Experimental Research.

[ref-71] Wang H, Liu YY, Pei ZG, Liang JF, Ding XS (2023). The influence of Tai Chi exercise on the subjective well-being in the aged: the mediating role of physical fitness and cognitive function. BMC Geriatrics.

[ref-72] Wang WC, Sawada M, Noriyama Y, Arita K, Ota T, Sadamatsu M, Kiyotou R, Hirai M, Kishimoto T (2010). Tai Chi exercise *versus* rehabilitation for the elderly with cerebral vascular disorder: a single-blinded randomized controlled trial. Psychogeriatrics.

[ref-73] World Health Organization (2005). Promoting mental health: concepts, emerging evidence, practice: a report of the World Health Organization, Department of Mental Health and Substance Abuse in collaboration with the Victorian Health Promotion Foundation and the University of Melbourne.

[ref-74] World Health Organization (2020). WHO guidelines for physical activity and sedentary behaviour. https://www.who.int/publications/i/item/9789240015128.

[ref-75] Wu X, Xue WG, Fang J, Wang JX, Fan XL, Ma HX, Liu Z, Ye CD, Duan JX (2017). Baduanjin of movements on the risk of falls in community elderly people for 60 cases. Chinese Medicine Modern Distance Education of China.

[ref-76] Xiao Z, Cruz M, Hojo E, Eungpinichpong W, Wang XZ, Xiao L, Chatchawan U, Hu Y, Roberts N (2023). The benefits of Shuai Shou Gong (SSG) demonstrated in a Randomised Control Trial (RCT) study of older adults in two communities in Thailand. PLOS ONE.

[ref-77] Xie JY, Guo JD, Wang B (2024). Comparing the effectiveness of five traditional Chinese exercises in improving balance function in older adults: a systematic review and Bayesian network meta-analysis. PeerJ.

[ref-78] Ying YA, Shen CZ, Wang XM (2019). Effects of Baduanjin on sleep quality and anxiety in older adults with insomnia living in senior care institutions. Electronic Journal of Clinical Medical Literature.

[ref-79] Zheng S, Kim C, Lal S, Meier P, Sibbritt D, Zaslawski C (2018). The effects of twelve weeks of Tai Chi practice on anxiety in stressed but healthy people compared to exercise and wait-list groups—a randomized controlled trial. Journal of Clinical Psychology.

