# Supplementary material for: Psychological health outcomes of traditional Chinese exercises in older adults: a meta-analysis of randomized controlled trials
Source: PeerJ. 2026 Feb 11;14:e20773. doi: 10.7717/peerj.20773 (PMC12906261; doi:10.7717/peerj.20773)
Supplement: Supplemental Information 2 [file peerj-14-20773-s002.docx]

## Systematic Review and/or Meta-Analysis Rationale

For systematic reviews / meta-analyses, authors need to provide the following information:

1.The rationale for conducting the systematic review / meta-analysis.

**Reply:** In recent years, an increasing number of systematic reviews and meta-analyses have examined the health benefits of traditional Chinese exercises (TCEs). However, most of the reviews and meta-analyses have just focused on their effects on physical function in older adults. This study conducts a meta-analysis of existing randomized controlled trials (RCTs) to investigate the effects of TCEs on psychological health outcomes.

2.The contribution that it makes to knowledge in light of previously published related reports, including other meta-analyses and systematic reviews.

**Reply:** As far as we know, no prior meta-analysis has comprehensively evaluated the effects of TCE interventions on a broad range of psychological health outcomes in older adults. This study addressed this gap by including multiple psychological outcomes, such as depression, anxiety, subjective well-being, general self-efficacy, and self-esteem, and by synthesizing evidence exclusively from RCTs, which represent the highest level of evidence.
